# Repeated sprint training: The effects of session volume on acute physiological, neuromuscular, perceptual and performance outcomes in athletes

**DOI:** 10.1002/ejsc.12217

**Published:** 2024-11-25

**Authors:** Fraser Thurlow, Shaun J. McLaren, Andrew Townshend, Matthew Morrison, Nicholas Cowley, Jonathon Weakley

**Affiliations:** ^1^ School of Behavioural and Health Sciences Australian Catholic University Brisbane Queensland Australia; ^2^ Sports Performance, Recovery, Injury and New Technologies (SPRINT) Research Centre Australian Catholic University Brisbane Queensland Australia; ^3^ Newcastle Falcons Rugby Club Newcastle Upon Tyne UK; ^4^ Department of Sport and Exercise Sciences Manchester Metropolitan University Institute of Sport Manchester UK; ^5^ Carnegie Applied Rugby Research (CARR) Centre Carnegie School of Sport Leeds UK

**Keywords:** cardiorespiratory, fitness, periodisation, team sport, training load

## Abstract

We examined the effects of repeated sprint training (RST) session volume on acute physiological, neuromuscular, perceptual and performance outcomes. In a randomised, counterbalanced, and crossover design, 14 healthy and trained male and female athletes (age: 23 ± 3 years) completed two sets of 10 × 40 m (10 × 40), 5 × 40 m (5 × 40), 10 × 20 m (10 × 20) and 5 × 20 m (5 × 20) sprints with 30 s rest between repetitions and 3 min rest between sets for all protocols. Average and peak heart rate, average oxygen consumption (VO_2_), time >90% of maximal oxygen consumption (VO_2max_), differential ratings of perceived exertion (RPE), session‐RPE training load (sRPE‐TL), percentage sprint decrement (S_dec_), acceleration load and distance >90% of maximal sprint speed were recorded during each session. Neuromuscular performance (i.e. countermovement jump, lower‐limb stiffness and isometric hamstring strength) were measured post‐session, 24 h, and 48 h and compared to pre‐session. A univariate analysis of variance was used to compare within‐ and between‐protocol differences. To aid data interpretation, all effects were expressed as an effect size and accompanied by probability values (*p*_*MET*_). The 10 × 40 protocol induced the greatest training load compared to all other protocols (*p*_*MET*_ < 0.05), including *moderate* to *very large* differences in breathlessness RPE, *large* differences in S_dec_ and time >90% VO_2max_ and *very large* differences in sRPE‐TL. The 5 × 20 protocol induced the lowest training load compared to all other protocols (*p*_*MET*_ < 0.05), including *moderate* to *large* differences in sRPE‐TL and leg muscle RPE. Heart rate, VO_2_, sRPE‐TL, leg muscle RPE and S_dec_ were similar between 5 × 40 and 10 × 20 (*p*_*MET*_ < 0.05), but the acceleration load was greater for 10 × 20 when compared to 5 × 40 (*p*_*MET*_ < 0.001), and this difference was *large*. Changes in neuromuscular performance across all timepoints and all protocols were unclear. Larger session volumes increase the demands of RST and by manipulating volume, sprint distance and the number of repetitions, practitioners can alter the internal and external training load.

## Highlights

- The 10 × 40 protocol (i.e. 800 m volume) induced the greatest physiological and perceptual demands, which was demonstrated by a session average heart rate and VO_2_ of 89% ± 3% and 72% ± 8% of max, respectively, whereas differential RPE were rated hard to very hard on average.
- Performing 10 repetitions did not substantially increase the average VO_2_ demands when sprint distance was matched (e.g. 10 × 40 vs. 5 × 40). Rather than prescribing high repetition protocols, practitioners are encouraged to increase sprint distance if a greater aerobic stimulus is desired.
- There were no differences in the heart rate and VO_2_ recovery between the 2nd and 3rd minute of inter‐set recovery. Therefore, to reduce session duration while providing adequate recovery of cardiorespiratory function, 2‐min passive rest periods can be prescribed.

## 1 INTRODUCTION

Repeated sprint training (RST) is an effective training method that can improve a range of physical qualities, including speed, aerobic fitness and intermittent running performance (Taylor et al., 2015; Thurlow, Huynh, et al., 2023). Sessions are typically 10−20 min in duration and involve maximal effort, short duration sprints (≤10 s) and interspersed with brief recovery times (≤60 s; Girard et al., 2011; Thurlow, Weakley, et al., 2023). There are several situations where RST can be applied in an athlete's training programme, such as the specific preparation stage of pre‐season where an increase in intensity is often implemented (Bompa et al., 2019). Alternatively, it could be used to maintain physical qualities during the playing season or as part of late‐stage rehabilitation. Recent evidence has demonstrated that RST induces substantial acute physiological demands, including considerable increases in blood lactate and mean and peak heart rate (Thurlow, Weakley, et al., 2023). However, these responses can be moderated by the manipulation of programming variables (Thurlow, Weakley, et al., 2023). For example, peak heart rate, blood lactate and sprint performance are maintained during RST when four repetitions are completed per set compared to six, but a 10‐m‐long repetition distance (40 vs. 30 m) can amplify these demands and increase fatigue (Thurlow, Weakley, et al., 2023). Thus, to ensure that the appropriate training load is imposed upon athletes, practitioners need to carefully consider the manipulation of programming variables.

One programming variable that has a large influence on the acute physiological demands of RST is session volume (i.e. repetition distance (*m*) × number of repetitions (*n*)). The volume of RST prescribed within the scientific literature typically ranges from 200 to 800 m per session and this appears to strongly influence the acute demands of RST (Taylor et al., 2015; Thurlow, Weakley, et al., 2023). For example, larger session volumes (≥800 m) cause a peak heart rate of ≥90% of the maximal heart rate (HR_max_) (Figueira et al., 2021; Paulauskas et al., 2020), which may help to maximise aerobic adaptations with high‐intensity training (Buchheit et al., 2013a). Additionally, Dupont et al. (2005) showed that players could reach maximal oxygen consumption (VO_2max_) when a session volume of 600 m was implemented. Despite its acknowledged importance within the scientific literature (Thurlow, Weakley, et al., 2023), evidence of the effects of different session volumes on the acute physiological demands of RST is lacking. Studies have manipulated repetition distance (Alemdaroğlu et al., 2018; Dellal et al., 2015) or the number of repetitions (Gharbi et al., 2014) but never compared both while standardising all other programming variables. This information could provide coaches with strategies to amplify the training stimulus, which would be expected to enhance subsequent physiological adaptation.

Athletes require regular exposure to sprinting within the training environment to effectively prepare them for high‐speed demands of competition (Gabbett, 2016; Malone et al., 2017; Oakley et al., 2018). In team sports, such as soccer and Australian Rules Football, players can achieve mean sprint (>23 km⋅h^−1^) distances of 337 and 571 m per game, respectively (Coutts et al., 2010; Di Salvo et al., 2007). RST can provide controlled doses of near‐to‐maximal speed running (Edouard et al., 2019; Malone et al., 2017; Mendiguchia et al., 2020), but coaches need to consider the optimal volume of maximal velocity exposure so that excessive or insufficient volumes of sprint training do not hinder performance (Gabbett, 2016; Malone et al., 2017). There can be a considerable neuromuscular demand during RST (Thurlow, Weakley, et al., 2023) and this may be exacerbated by the prescription of larger session volumes (Buchheit et al., 2013b). Previous studies have shown that greater RST volumes reduce countermovement jump (CMJ) performance and acute knee flexor strength (Baumert et al., 2021; Clifford et al., 2016; Timmins et al., 2014). Furthermore, these reductions may persist for up to 48 h post‐exercise (Baumert et al., 2021; Woolley et al., 2014). Given the possible effects of volume on fatigue and recovery time course, it is important to understand the effects of this programming variable as well as the relationship between the two individual factors that constitute session volume (i.e. repetition distance and the number of repetitions). Therefore, the aims of our investigation were to examine the effects of manipulating session volume on acute physiological, perceptual and performance demands during RST and the recovery time course of neuromuscular performance and determine whether repetition distance or the number of repetitions has a greater effect on the acute demands and the recovery time course.

## 2 MATERIALS AND METHODS

### 2.1 Experimental approach to the problem

A randomised, crossover and counterbalanced design (Latin square) was used to compare the effects of four different RST protocols. Heart rate, VO_2_, differential ratings of perceived exertion (dRPE), percentage sprint decrement (S_dec_), acceleration load and volume of sprinting >90% of maximal sprint speed (MSS) were recorded during each session. Lower‐limb stiffness, CMJ performance and isometric hamstring strength were measured immediately pre‐ and post‐session, 24 h and 48 h, whereas session ratings of perceived exertion (sRPE) were also recorded post‐session. The study was conducted over 4 weeks for each participant and involved one RST session per week performed on Monday and two follow‐up testing sessions 24 and 48 h afterwards. In total, the athletes attended 13 sessions (i.e. familiarisation and 12 testing sessions). The RST protocols were prescribed with different combinations of the number of repetitions and sprint distance (i.e., 5 or 10 repetitions and 20 or 40 m distance), whereas all other programming variables were fixed across all sessions (Table 1). Together, the training protocols represent the typical range of session volumes (200−800 m) used in research and practice (Thurlow et al., 2023a, 2023b). Furthermore, it is common for coaches to progress session volume from 200 to 800 m across the course of training program.

**TABLE 1:** Prescription of the repeated sprint training protocols.

Protocol	Sets × Reps	Sprint distance (m)	Inter‐rep rest time (s)	Inter‐set rest time (s)	Rest mode	Prescribed volume (m)
10 × 40	2 × 10	40	30	180	Passive	800
10 × 20	2 × 10	20	30	180	Passive	400
5 × 40	2 × 5	40	30	180	Passive	400
5 × 20	2 × 5	20	30	180	Passive	200

### 2.2 Subjects

Fourteen trained athletes, training at least three times per week with the purpose of competing at a local level or higher, were recruited from a university to take part in our study. The physical characteristics of the athletes are presented in Table 2. Before initiating the study, athletes were informed of the procedures, risks and benefits and signed an institutionally approved informed consent form. All athletes were injury‐free for at least 3 months before the study and no injuries or dropouts occurred during the study. The study protocol adhered to the declaration of Helsinki and was approved by a university institutional review board (ethics number: 2021‐244H).

**TABLE 2:** Physical characteristics of the athletes.

	Age (years)	Height (cm)	Weight (kg)	VO_2max_ (mL⋅kg^−1^⋅min^−1^)
Males (*n* = 10)	24 ± 4	182 ± 9	83 ± 10	57 ± 6
Females (*n* = 4)	22 ± 1	169 ± 6	62 ± 3	45 ± 2

### 2.3 Procedures

All athletes attended a familiarisation session one week before the commencement of the study where they signed consent forms, practised all testing procedures and had their height measured (Seca Alpha stadiometer, model 213). Additionally, the athletes completed a graded exercise test on a motorised treadmill (T22.1, Vertex Fitness) with respiratory gas analysis (K5, COSMED, Rome, Italy) to determine their VO_2max_ (mL·kg·min^−1^) and HR_max_. All testing took place at the same time of day (±1 h) to minimise any potential influence of diurnal or circadian variation. In the day preceding the familiarisation sessions, each RST session as well as between each session and the follow‐ups, the athletes were instructed to refrain from strenuous exercise involving the leg muscles (e.g., running, resistance training, sports activity) and from consuming alcohol. The athletes were also instructed to abstain from the consumption of food and beverage other than water within 2 h of each session, and the consumption of caffeine 6 hours before each session. In addition to these restrictions, the athletes were also asked to maintain their usual nutritional habits during the intervention period. The sprints were performed on a grass sports oval, under similar environmental conditions (21°C−28°C and 54%–78% humidity).

**FIGURE 1:**
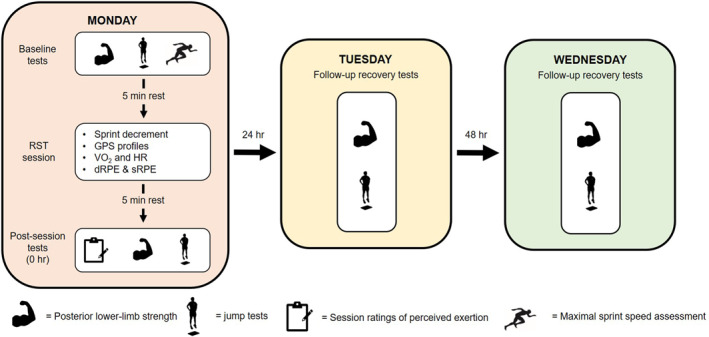
The experimental procedures for one repeated sprint training session and its follow‐up sessions. This design is repeated for each of the four repeated‐sprint training protocols, which are separated by 1 week. dRPE, differential ratings of perceived exertion; GPS, global positioning system; HR, heart rate; RST, repeated‐sprint training and VO_2_, oxygen consumption.

The experimental procedures for each RST session and its follow‐up recovery sessions can be seen in Figure 1. At the beginning of each RST session, the athletes performed the same standardised warm‐up (warm‐up A) consisting of a series of dynamic movements performed over a distance of 10 m (e.g. walking lunges, heel sweeps and A‐skips). Baseline testing was then performed in the following order: (1) a unilateral isometric strength test of the hamstring muscles, (2) a CMJ test and (3) a double‐leg hopping test. Following the baseline tests, the athletes performed an additional warm‐up (warm‐up B), which involved 4 × 40‐m strides at an estimated 50%, 70%, 80% and 90% of maximal speed. Between each effort, the athletes slowly walked back to the starting point. Following the final stride, the athletes performed 1 × 40 m maximal sprint to determine their peak velocity for that day, which was identified via a 10‐Hz global position system (GPS; Apex, STATSports) that was fitted within a vest on the athlete's upper thoracic region (Crang et al., 2021, 2022). The athletes were then provided a 5‐min rest before beginning the RST session and during this rest period, they were fitted with the same automated, wearable, gas analysis system as used during the graded exercise test. Heart rate, VO_2_, repetition times and GPS data were recorded throughout the RST session. Differential ratings of perceived exertion were recorded at the end of set one and set two, whereas session ratings of perceived exertion (sRPE) were recorded 5 min following the end of the RST session, with both having been used extensively throughout the literature to quantify the perception of effort that athletes experience during exercise (Dudley et al., 2023; Weakley et al., 2019, 2022a). Although we acknowledge that the collection of sRPE has typically taken 30 min post‐session (McLaren et al., 2016, 2017), as follow‐up tests were conducted 5 min post‐session, sRPE was taken immediately before these. The post‐session testing was conducted in the same order as baseline testing. For the 24 and 48 h follow‐up sessions, the athlete's perceived muscle soreness was recorded at the beginning of the session. The same standardised dynamic warm‐up was performed (warm‐up A) before commencing testing. Athletes wore the same footwear and were fitted with the same GPS unit across each session (Crang et al., 2021).

#### 2.3.1 Repeated‐sprint training

The RST protocols are shown in Table 1. A 3‐min rest period was provided between sets, from the end of set one (i.e. the moment the athlete crossed the finish line after the final sprint) to the start of the first sprint in set two. During the inter‐set recovery period, athletes decelerated and walked back to the starting point, where they sat on a chair until the beginning of set two. Athletes started each sprint in a standing start position with their foot 0.3 m behind the first timing gate (Weakley, McCosker, et al., 2022). A 10‐s warning and a 3‐s countdown was provided for each repetition. Athletes were instructed to give maximal effort and sprint through the finish line. Loud verbal encouragement was given to all athletes during each repetition. During the recovery period between sprints, athletes decelerated and walked back to the starting point. Two sets of single‐beam timing gates (TCi, Brower Timing Systems) were used that worked in both directions, which allowed the athletes to start each sprint at the end they finished the previous sprint. The timing gates were used to determine the sprint times of each repetition, whereas GPS was used to determine the acceleration load (i.e. dynamic stress load during acceleration: the sum of the magnitude of the tri‐axial accelerometer data above 2G, raised to a body load factor and scaled to manageable values) and the volume of sprinting >90% of each individuals MSS during each session (Murray et al., 2018). The fastest peak velocity derived from the GPS achieved during baseline testing (1 × 40 m maximal sprint) for each athlete on each training day was used as the reference MSS. This approach allowed daily individual fluctuations in sprint performance to be accounted for. To calculate the decline in sprint speed across each set, S_dec_ was used (Fitzsimons et al., 1993).While S_dec_ has been shown to be less reliable than best and average times for detecting changes in performance (Impellizzeri et al., 2008), it is the most ecologically valid index to quantify fatigue during RST (Glaister et al., 2008). It was calculated as follows:

$$S_{\text{dec}}=\left(100\times \left(\text{total}\;\text{sprint}\;\text{time}/\;\text{ideal}\;\text{sprint}\;\text{time}\right)\right)-100$$

where total sprint time represents the sum of sprint times from all sprints and ideal sprint time represents the number of sprints multiplied by the fastest sprint time. The average of both sets for S_dec_ was used for analysis.

#### 2.3.2 Hamstring strength

Maximal sprinting induces a high degree of stress and strain on the hamstring muscles (Schache et al., 2012; Thelen et al., 2005; Timmins et al., 2014), with previous evidence demonstrating that declines in hamstring strength may persist for several days following repeated‐sprints (Baumert et al., 2021; Timmins et al., 2014). To monitor changes in hamstring strength, an isometric assessment was performed on a portable force plate (ForceDecks, VALD Performance). Isometric tests result in little or no structural muscle damage (Faulkner et al., 1993; Lieber et al., 1991; McCall et al., 2015; Nosaka et al., 2002); thus they are useful to assess muscle function between recovery timepoints. The test was performed on the athletes' dominant limb at knee angles of 90° and 30°. These angles were chosen because the biceps femoris has been shown to be maximally activated between 15° and 30° of knee flexion (from full knee extension), whereas the semimembranosus and semitendinosus were maximally activated between 90° and 105° of knee flexion (Onishi et al., 2002). The tests have previously demonstrated good−high levels of reliability (intraclass correlation coefficient = 0.86–0.95 and typical error as a coefficient of variation = 4.3%–6.3%; McCall et al., 2015). To complete this test, athletes laid on their back on a mat, with the non‐working leg resting flat on the floor and the heel of the working leg positioned on the force plate, which was placed on a firm box. The knee angle of the athletes' working leg was set using a goniometer (EZ Read Jamar, Patterson Medical). The athlete was instructed to push the heel of their working leg into the force platform as hard as possible as though they were trying to perform a hamstring curl, without lifting their hips, hands or head off the mat. The contraction was performed for 3 s and repeated three times at each angle with 30 s rest between trials. The highest peak force (*N*) from the three trials was recorded for analysis. Investigators ensured strict adherence to the technique by pressing the athletes' hips to the floor during each repetition and giving loud verbal encouragement throughout to ensure maximal effort.

#### 2.3.3 Jump testing

The CMJ test is a suitable athlete monitoring method for the detection of neuromuscular fatigue (Gathercole et al., 2015). It has been recommended that a battery of CMJ variables be used rather than jump height alone (Gathercole et al., 2015). Therefore, metrics were chosen which have acceptable intra‐day and inter‐day reliability (<10% coefficient of variation) are sensitive to changes in neuromuscular function and provide a more detailed analysis that reflects changes in CMJ output and strategy (Cormack et al., 2008; Gathercole et al., 2015; Weakley, Black, et al., 2022). Jump testing was performed on the same portable force plates as the hamstring strength assessment. For the CMJ, athletes began in a standing position and were instructed to jump as high as possible while keeping their hands on their hips. The depth of the countermovement was self‐selected by the athlete (Cormack et al., 2008). Three maximal trials were performed with a 30‐s rest between each effort. Jump height was analysed using the impulse–momentum method as it gives the most accurate result (Linthorne, 2001) (Linthorne, 2001), with jump initiation detected as a change of 20 N from the start of the movement. The trial with the greatest jump height was used for analysis.

The double‐leg hopping test has been previously used with athletic populations to provide a measure of leg stiffness and consisted of sub‐maximal rebounding at 2.5 Hz (150 bpm) (Dalleau et al., 2004; Leduc et al., 2020; Oliver et al., 2015). This frequency possesses the highest reliability and allows participants to maintain a consistent hopping pace while acting in a true spring‐mass manner (Lloyd et al., 2009). Athletes completed one trial of 20 consecutive hops with hopping frequency controlled by a digital metronome (TempoPerfect, version 4.07, HCH Software). The first and last five hops were discarded, with an average of the hops 6−15 used for analysis. Leg stiffness was calculated through Dalleau's equation (50):

$$\text{Leg}\;\text{stiffness}=\frac{M\times \pi \left(Ft+Ct\right)}{{Ct}^{2}\left(\left(Ft+\frac{Ct}{\pi }\right)-\left(\frac{Ct}{4}\right)\right)}$$

where M is the mass (kg), Ft is the flight time (s) and Ct is the contact time (s).

#### 2.3.4 Perceptual measures

Differential ratings of perceived exertion can enhance the accuracy of the internal load measurement by better discriminating between central (e.g. uptake and transport of oxygen and central nervous system) and peripheral exertions (e.g. neuromuscular, musculoskeletal and metabolite characteristics; McLaren et al., 2016). Immediately following the completion of each RST set, athletes provided dRPE for breathlessness (RPE‐B) and leg exertion (RPE‐L) by considering verbal anchors on a Borg CR100 Scale^®^ (Borg, 2010). Athletes were instructed that their ratings should reflect the perceptions of effort experienced for the preceding set only (McLaren et al., 2020) and they were informed about the definition of perceived exertion and its scaling, including the importance of separating rating of perceived exertion from other exercise‐related sensations such as pain, discomfort and fatigue (McLaren et al., 2020). Instructions were also given to athletes on how to appraise dRPE, such that RPE‐B depends mainly on the breathing rate and/or heart effort and RPE‐L depends mainly on the strain and exertion in the leg muscles (McLaren et al., 2020). The average of both sets for dRPE was used for analysis. Five minutes after the RST session, athletes also provided a global sRPE by considering verbal anchors on a modified version (Foster et al., 2001) of the Borg CR10 Scale^®^ (Borg, 2010), which was multiplied with the session duration to determine sRPE‐training load (sRPE‐TL; Foster et al., 2001).

#### 2.3.5 Oxygen consumption and heart rate

During the familiarisation session, athletes completed a graded exercise test on a motorised treadmill (T22.1, Vertex Fitness) with respiratory gas exchange data collected via a portable metabolic system and heart rate measured using a chest strap monitor (HRM‐Dual, Garmin Australasia Pty Ltd), which was integrated with the metabolic system. To become familiarised with the portable metabolic system and Hans Rudolph face mask, participants wore these apparatuses during the warm‐up, which consisted of 3−5 min of running at a self‐selected pace and any other preparatory exercise of their choosing. Depending on the athlete's fitness level, the test then began at a speed between 6 and 10 km⋅h^−1^. Each stage lasted for 2 min and increased by 2 km⋅h^−1^ for the first three stages, after which the speed was maintained while the gradient increased by 2%, every 2 min until the participant reached volitional exhaustion, which was achieved within 10−14 min for all participants (Beltz et al., 2016). Analysis of the graded exercise test was performed by removing erroneous fluctuations in raw data and then averaging VO_2_ into 15 s time bins, with the highest value used to determine the athlete's VO_2max_.

For the RST sessions, the heart rate and respiratory gas exchange were continuously recorded from the start of the first repetition to 30 s following the final repetition, using the same equipment as the graded exercise test. Erroneous fluctuations in raw data were removed if they were considered to be higher or lower than physiologically possible. Heart rate and VO_2_ data were averaged for each repetition, set and the overall RST session (excluding the inter‐rest recovery period). Peak heart rate was identified from the highest value during each set and the overall RST session. Heart rate and VO_2_ data from the inter‐set rest period were also analysed, which included the value at the end of set one (from the moment the athlete passed the timing gate after the last sprint), and the decline after 1, 2 and 3 min. For the analysis of VO_2_ during the inter‐set rest period, a 15‐s average was used, so that the VO_2_ decline at 1 mi, 2 and 3 min were recorded from 45 to 60 s, 105 to 120 s and 165 to 180 s, respectively.

### 2.4 Statistical analyses

The mean ± standard deviation (SD) was calculated for all outcomes. The Shapiro–Wilk test confirmed all variables were normally distributed. A univariate analysis of variance (ANOVA) was used to compare between protocol differences in physiological, perceptual and sprint performance outcomes and within protocol differences (pre–post, pre‐24 h and pre‐48 h) in recovery outcomes. To aid with data interpretation, all effects were expressed as an effect size (ES) by dividing the estimate and its confidence limit (CL) by the pooled between‐subject SD of each protocol (subsequently adjusted for small sample bias; Hedges G) and day‐to‐day variability (Bernards et al., 2017). Values of 0.2, 0.6, 1.2 and 2.0 represent thresholds for small, moderate, large and very large differences for the standardised difference in means (Hopkins, 2002). A difference was declared when the upper and lower confidence interval fell entirely or predominantly outside the non‐substantial region (i.e. outside −0.2 to 0.2). When this was visually apparent, a minimum effects test (MET) was used to provide a probabilistic description of the CL's disposition relative to the threshold for a non‐substantial effect. Given that this study was not powered for definitive conclusions, we elected to present probability values for the one‐sided tests (*p*_MET_) as continuous estimates only, rather than declaring a fixed alpha level representing ‘practical significance’. Data analysis was conducted using the SPSS 29 program for Windows (SPSS, Inc.).

## 3 RESULTS

**FIGURE 2:**
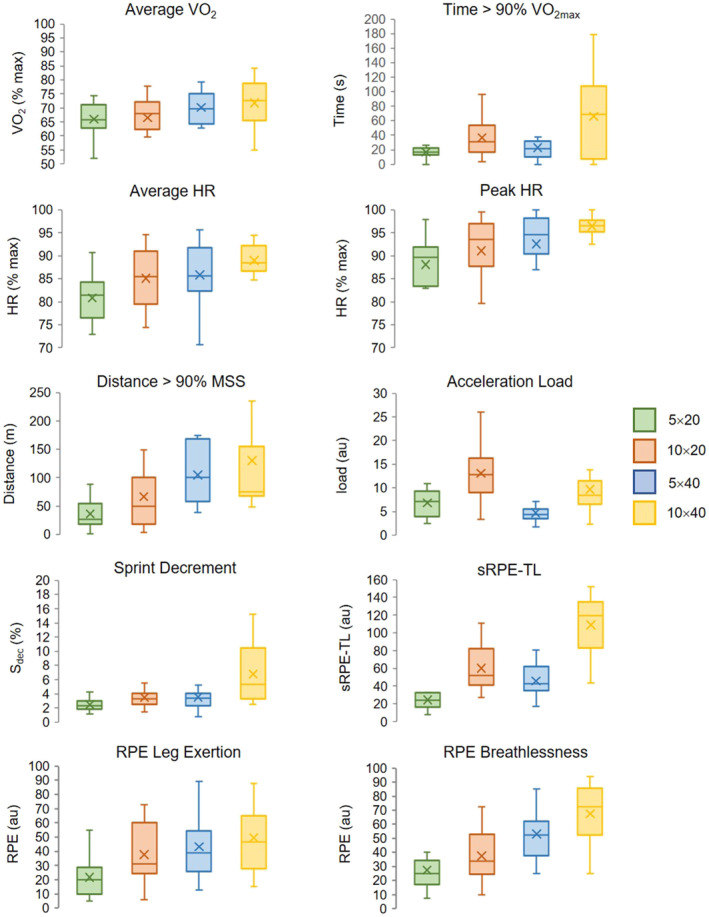
The acute demands of each RST protocol. Green = 5 × 20, orange = 10 × 20, blue = 5 × 40, yellow = 10 × 40 and × = mean. au, arbitrary units; HR, heart rate; RPE, rating of perceived exertion; S_dec_, percentage sprint decrement; sRPE‐TL, session RPE‐training load; VO_2_, oxygen consumption and VO_2max_, maximal oxygen consumption.

**FIGURE 3:**
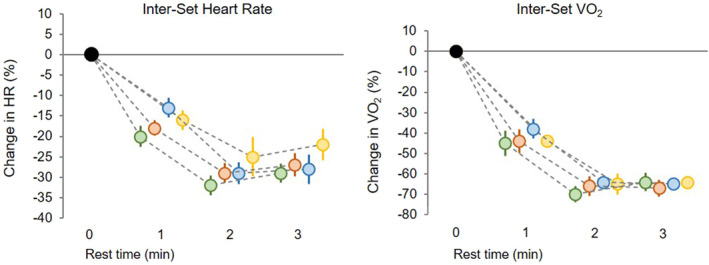
Changes (mean ± 90% confidence limit) in the heart rate and oxygen consumption across the inter‐set recovery period between set one and set two for each repeated‐sprint training protocol. green = 5 × 20, orange = 10 × 20, blue = 5 × 40 and yellow = 10 × 40. The inter‐set recovery period is the time between the end of the last sprint repetition in set one (0 min) and the start of the first sprint repetition in set two (3 min).

Descriptive data on the acute physiological, perceptual and performance demands of each RST protocol are presented in Figure 2 and Supplementary Digital Contents 1 and 2. Additionally, Figure 3 displays the change in heart rate and VO_2_ across the inter‐set recovery period for each RST protocol.

### 3.1 Training load

#### 3.1.1 Physiological and perceptual measures (internal training load)

Session average heart rate was higher for 10 × 40 and 5 × 40 when compared to 5 × 20 (*p*_*MET*_ = 0.002 and 0.059, respectively), and these differences were *large* and *moderate* (ES: 1.38 ± 0.65 and 0.80 ± 0.64, respectively). Additionally, the session peak heart rate was higher for 10 × 40 when compared to 5 × 20, and this difference was *moderate* (ES: 1.10 ± 0.83 and *p*_*MET*_ = 0.011). Time >90% VO_2max_ was greater for 10× × 40 when compared to 5 × 40 (*p*_*MET*_ = 0.002) and 5 × 20 (*p*_*MET*_ < 0.001), and these differences were *large* (ES: 1.29 ± 0.62 and 1.47 ± 0.63, respectively).

Differential RPE‐L was greater for 10 × 40 (*p*_*MET*_ = 0.001), 5 × 40 (*p*_*MET*_ = 0.013) and 10 × 20 (*p*_*MET*_ = 0.063) when compared to 5 × 20, and these differences were *large, moderate and moderate* (ES: 1.37 ± 0.63, 1.06 ± 0.63 and 0.78 ± 0.63, respectively). RPE‐B was greater for 10 × 40 when compared to 5 × 40 (*p*_*MET*_ = 0.064), 10 × 20 (*p*_*MET*_ < 0.001) and 5 × 20 (*p*_*MET*_ < 0.001), and these differences were *moderate*, *large* and *very large* (ES: 0.79 ± 0.64, 1.64 ± 0.64 and 2.19 ± 0.64). Furthermore, RPE‐B was greater for 5 × 40 when compared to 10 × 20 (*p*_*MET*_ = 0.046) and 5 × 20 (*p*_*MET*_ = 0.001), and these differences were *moderate* and *large* (ES: 0.85 ± 0.64 and 1.41 ± 0.64, respectively).

Session RPE‐TL was greater for 10 × 40 when compared to 5 × 40 (*p*_*MET*_ < 0.001), 10 × 20 (*p*_*MET*_ < 0.001) and 5 × 20 (*p*_*MET*_ < 0.001), and these differences were *very large* (ES: 2.59 ± 0.63, 2.00 ± 0.63 and ES: 3.47 ± 0.63, respectively). Session RPE‐TL was also greater for 5 × 40 (*p*_*MET*_ = 0.039) and 10 × 20 (*p*_*MET*_ < 0.001) when compared to 5 × 20, and these differences were *moderate* and *large* (ES: 0.88 ± 0.63 and 1.47 ± 0.63, respectively). All other comparisons of the internal training load were not definitive and can be found in Supplementary Digital Content 3 and 4.

#### 3.1.2 Performance measures (external training load)

Sprint decrement was greater for 10 × 40 when compared to 5 × 40 (*p*_*MET*_ = 0.002), 10 × 20 (*p*_*MET*_ = 0.001) and 5 × 20 (*p*_*MET*_ < 0.001), and these differences were *large* (ES: 1.37 ± 0.64, 1.39 ± 0.64 and 1.79 ± 0.64, respectively). Session distance >90% MSS was greater for 10 × 40 when compared to 10 × 20 (*p*_*MET*_ = 0.029) and 5 × 20 (*p*_*MET*_ = 0.001), and these differences were *moderate* and *large* (ES: 0.94 ± 0.64 and 1.38 ± 0.63, respectively). Additionally, session distance >90% MSS was greater for 5 × 40 when compared to 5 × 20 (*p*_*MET*_ = 0.018), and this difference was *moderate* (ES: 1.05 ± 0.66). Session acceleration load was greater for 10 × 40 when compared to 5 × 40 (*p*_*MET*_ = 0.013), and this difference was *moderate* (ES: 1.07 ± 0.64). Furthermore, the session acceleration load was greater for 10 × 20 when compared to 5 × 40 (*p*_*MET*_ < 0.001), and this difference was *large* (ES: 1.83 ± 0.63). All other comparisons of the external training load were not definitive and can be found in Supplementary Digital Content 5.

### 3.2 Recovery measures

**FIGURE 4:**
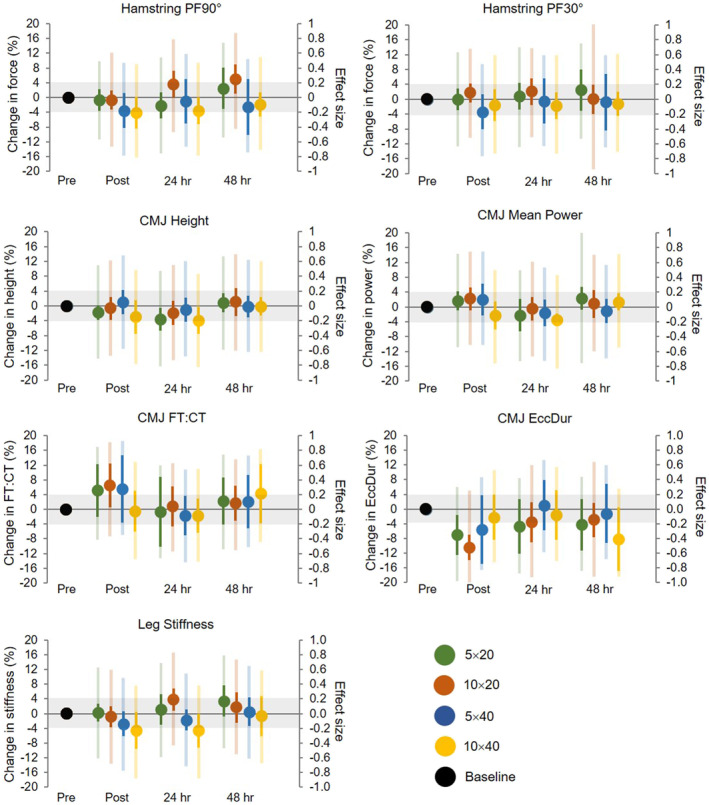
The recovery time course of neuromuscular performance within each repeated sprint training protocol. Green = 5 × 20, orange = 10 × 20, blue = 5 × 40 and yellow = 10 × 40. Dark error bar represents the 90% confidence limit for the percent change in performance; shaded colour represents the 90% confidence limit for the standardised difference and grey shaded zone represents a trivial effect. CMJ, countermovement jump; EccDur, eccentric duration; FT:CT, flight‐time to contraction‐time ratio; PF90°, peak force at 90° of knee flexion and PF30°, peak force at 30° of knee flexion.

The effects of RST on the recovery time course of neuromuscular performance are presented in Figure 4 and Supplementary Digital Content 6. Changes in neuromuscular performance across all timepoints and all protocols were unclear.

## 4 DISCUSSION

Our study aimed to examine the effects of manipulating session volume on acute physiological, perceptual and performance demands during RST and the recovery time course of neuromuscular performance and determine whether repetition distance or the number of repetitions has a greater effect on the acute demands and the recovery timecourse. The 10 × 40 protocol induced the greatest physiological and perceptual demands, which was demonstrated by a session average heart rate and VO_2_ of 89 ± 3% and 72 ± 8% of max, respectively, whereas dRPE were rated hard to very hard on average. Additionally, the 10 × 40 protocol also had the greatest S_dec_ and incurred a sRPE‐TL that was higher than all other protocols by a *very large* magnitude. Conversely, the acute demands of the 5 × 20 protocol were considerably less than all other protocols. When session volume was matched at 400 m, the internal training load was similar, but the acceleration load was greater for the 10× × 20 protocol, whereas sprint volume (>90% MSS) was higher for the 5 × 40 protocol. Across all protocols, there was substantial inter‐individual variation in neuromuscular function, and subsequently, the return to baseline of neuromuscular performance was unclear. The findings from our investigation demonstrate that larger session volumes increase the acute demands of RST and by manipulating volume, sprint distance and the number of repetitions, practitioners can alter the internal and external training load.

This is the first investigation to directly examine time above 90% of VO_2max_ during RST, which has been suggested to be an important stimulus to elicit maximal cardiovascular and peripheral adaptations during high‐intensity interval training (Billat, 2001; Laursen et al., 2002; Midgley et al., 2006). We found that 66 ± 56 s was spent above 90% of VO_2max_ during the 10 × 40 protocol, which was greater than the other protocols by a *large* magnitude. In agreement with evidence by Gaitanos et al. (1993), performing more repetitions in succession increases aerobic metabolism. This occurs due to extended session durations and increased within session fatigue as demonstrated by a greater S_dec_ for the 10 × 40 protocol. However, in comparison to other high‐intensity conditioning methods, such as short‐ and long‐intervals, which may elicit >10 min above 90% of VO_2max_ (Buchheit et al., 2013b), time above 90% of VO_2max_ was low for all four RST protocols. Therefore, the strategy of implementing high repetition protocols to increase time above 90% of VO_2max_ during RST is likely futile because increases will be relatively minimal. Furthermore, compared to sets prescribed with five repetitions, performing 10 repetitions did not substantially increase the average VO_2_ demands when sprint distance was matched (e.g. 10 × 40 vs. 5× × 40). Rather than prescribing high repetition protocols, practitioners are encouraged to increase sprint distance or manipulate other RST variables, such as rest time (Thurlow, Weakley, et al., 2023), to enhance the aerobic stimulus. Future research should investigate the acute and chronic effects of manipulating sets and repetitions within volume‐matched protocols (i.e. two sets of 10 repetitions vs. four sets of five repetitions) as this could also be an effective strategy to augment physiological responses.

The moderating effects of programming variables on RST have previously suggested that sprint distance has a substantial influence on physiological demands (Thurlow, Weakley, et al., 2023). Our present investigation lends further support to this premise, showing that 40 m sprints caused greater VO_2_ and heart rate compared to 20 m sprints, although this was only definitive for an average heart rate. Additionally, a greater volume of sprinting above 90% of MSS was achieved with 40 m sprints. The 10 × 40 and 5 × 40 protocols elicited over 100 m of maximal sprinting per session compared to 67 ± 64 m and 36 ± 27 m for the 10× × 20 and 5 × 20 protocols, respectively. Sprint training above 90% of MSS has been proposed as a key component of hamstring injury risk management (Edouard et al., 2023), and our findings suggest that the prescription of RST with a repetition distance of 40 m can provide a concurrent sprint and physiological stimulus that is substantial. Alternatively, the acceleration load was increased during the 20‐m sprint protocols, which would be attributed to the athlete's accelerating faster with greater horizontal propulsive force over the short distance and more gradually over the 40‐m distance. Considering the importance of acceleration ability in intermittent sports (Johnston et al., 2014, 2018; Little et al., 2005), RST protocols that emphasise acceleration may be a worthwhile component of a training program. Differential RPE were also lower in the 20‐m RST protocols compared to the 40‐m protocols by *moderate* to *large* magnitudes. Therefore, shorter sprint protocols may reduce the perceptual demand on athletes during RST and target the development of acceleration performance.

The 10 × 40 protocol tended to cause greater decrements in neuromuscular performance following the RST sessions, particularly for leg stiffness, CMJ height and CMJ mean power (Figure 3). However, given the width of the effect size confidence limits, these and all other recovery outcomes were unclear. The certainty in these results was affected by considerable inter‐individual variation, with athletes demonstrating a decrement, no change or potentiation of neuromuscular performance following RST. Furthermore, the training volumes may have been insufficient to induce consistent change in neuromuscular performance across the athletes, but these were considered to be the lower (200 m) and upper (800 m) limits of volume that are prescribed within applied training environments (Thurlow, Weakley, et al., 2023). While practitioners are encouraged to consider the individual fatigue responses to RST, future research may benefit from using more sensitive measures to detect neuromuscular fatigue and a larger sample size to form more definitive conclusions.

There are several practical applications from our findings that coaches can use to improve the prescription of RST. Session volumes of 200 m, prescribed as two sets of 5 × 20 m sprints, could be applied at the beginning of a RST program to introduce athletes to maximal acceleration, while limiting training load, before progressing to larger volumes such as 400 m. If 400 m of volume is implemented, prescribing this session as two sets of 5 × 40 m sprints will provide athletes with exposure to maximal sprinting (∼100 m > 90% of MSS), whereas the prescription of two sets of 10 × 20 m sprints will emphasise the acceleration load. To maximise the acute physiological and neuromuscular demands of RST, which may result in a greater stimulus for adaptation, larger session volumes (i.e. 800 m) are recommended and these are best achieved by implementing longer sprint distances (i.e. 40 m). Lastly, we administered 3 min inter‐set rest periods and found that there were no differences in the heart rate and VO_2_ recovery between the 2nd and 3rd minute mark (Figure 3). Therefore, during congested training sessions, to reduce session duration while providing adequate recovery of cardiorespiratory function, 2 min passive rest periods can be prescribed.

Our study provides evidence of the acute effects of RST volume, but it has some limitations. First, given the altered sprint distances across our protocols, we acknowledge that the work‐to‐rest ratios were subsequently different, and this would influence the recovery between sprints and subsequent physiological demands. However, the application of work‐to‐rest ratios in a sporting environment is logistically difficult because the time taken to perform each sprint varies between repetitions and athletes. Accordingly, standardised rest times were selected because these are more common within the scientific literature (Thurlow, Weakley, et al., 2023) and practical within real‐world training environments. Second, we used measures of neuromuscular recovery that are frequently implemented within sports settings, but recognise that there is no single ideal model to study fatigue (Cairns et al., 2005), and other disruptions to homoeostasis may have occurred. Additionally, we did not assess changes in eccentric hamstring strength or muscle activity. Considering the eccentric demands of sprint activity on the hamstring muscles (Mendiguchia et al., 2020), this may provide different results to our isometric hamstring strength assessment, which reduced the potential influence of testing fatigue on our recovery outcomes and is highly practical within sporting environments (McCall et al., 2015; O'Keefe, 2020). Lastly, because of the small sample size, this study provides an exploratory analysis rather than definitive findings.

## 5 CONCLUSION

The findings from our study demonstrate that larger session volumes increase the acute demands of RST. A session volume of 800 m induces the greatest aerobic stimulus but also causes substantially greater within‐session fatigue (i.e. S_dec_), sRPE‐TL and dRPE. When session volume is matched at 400 m, the physiological and perceptual demands are similar but the external training loads (i.e. acceleration load and volume > 90% MSS) are dependent on the sprint distance. A session volume of 200 m elicits a low physiological stimulus but could be useful to introduce or maintain exposure to maximal sprinting. Practitioners can use our findings to alter the acute training stimulus based on the aims of the training program.

## CONFLICT OF INTEREST STATEMENT

All authors declare that they have no conflicts of interest.

## Supporting information

Supporting Information S1
