# Supplementary material for: Repeated sprint training: The effects of session volume on acute physiological, neuromuscular, perceptual and performance outcomes in athletes
Source: Eur J Sport Sci. 2024 Nov 25;25(1):e12217. doi: 10.1002/ejsc.12217 (PMC11680557; doi:10.1002/ejsc.12217)
Supplement: Supplementary file 1 — Supporting Information S1 [file EJSC-25-e12217-s001.docx]

**Supplementary Digital Content 1.** Physiological demands of the repeated-sprint training sessions.

| **Outcome** |  | **Protocol** | | | | | | | | |
| --- | --- | --- | --- | --- | --- | --- | --- | --- | --- | --- |
|  |  | **10×40** | | **5×40** | | **10×20** | | | **5×20** | |
|  |  | Raw | % Max | Raw | % Max | Raw | % Max | Raw | | % Max |
| **HR_avg_**  **(b·min^-1^)** | **Set 1** | 171 ± 7 | 88 ± 4 | 164 ± 14 | 85 ± 7 | 161 ± 14 | 83 ± 7 | 153 ± 13 | | 79 ± 6 |
|  | **Set 2** | 175 ± 9 | 90 ± 4 | 169 ± 15 | 87 ± 7 | 168 ± 14 | 87 ± 7 | 160 ± 10 | | 82 ± 5 |
|  | **Session** | 173 ± 6 | 89 ± 3 | 166 ± 14 | 86 ± 7 | 165 ± 14 | 85 ± 7 | 157 ± 11 | | 81 ± 5 |
| **HR_peak_**  **(b·min^-1^)** | **Session** | 188 ± 7 | 96 ± 2 | 180 ± 18 | 93 ± 8 | 177 ± 18 | 91 ± 8 | 171 ± 16 | | 88 ± 7 |
| **VO_2avg_**  **(L⋅min^-1^)** | **Set 1** | 2.96 ± 0.74 | 72 ± 9 | 2.79 ± 0.62 | 68 ± 7 | 2.67 ± 0.61 | 65 ± 9 | 2.58 ± 0.63 | | 62 ± 6 |
|  | **Set 2** | 2.97 ± 0.82 | 72 ± 9 | 2.90 ± 0.64 | 70 ± 6 | 2.76 ± 0.68 | 67 ± 8 | 2.75 ± 0.67 | | 66 ± 6 |
|  | **Session** | 2.97 ± 0.78 | 72 ± 9 | 2.85 ± 0.63 | 69 ± 6 | 2.72 ± 0.64 | 66 ± 8 | 2.72 ± 0.60 | | 64 ± 6 |
| **T > 90% VO_2max_ (s)** | **Set 1** | 29 ± 26 | - | 12 ± 10 | - | 18 ± 16 | - | 6 ± 3 | | - |
|  | **Set 2** | 37 ± 34 | - | 11 ± 8 | - | 19 ± 14 | - | 10 ± 5 | | - |
|  | **Session** | 66 ± 59 | - | 23 ± 17 | - | 37 ± 28 | - | 16 ± 7 | | - |
| Abbreviations: HR_avg_ = average heart rate; HR_peak_ = peak heart rate; VO_2avg_ = average oxygen consumption; T > 90% HR_max_ = time (seconds) above 90% of maximal heart rate; T > 90% VO_2max_ = time (seconds) above 90% of maximal oxygen consumption. | | | | | | | | | | |

**Supplementary Digital Content 2.** Performance and perceptual demands of the repeated-sprint training sessions.

| **Outcome**  **measure** |  | **Protocol** | | | |
| --- | --- | --- | --- | --- | --- |
|  |  | **10×40** | **5×40** | **10×20** | **5×20** |
| **S_dec_ (%)** | **Set 1** | 6.3 ± 3.6 | 3.4 ± 1.9 | 3.4 ± 1.7 | 2.4 ± 1.0 |
|  | **Set 2** | 7.3 ± 5.1 | 3.6 ± 1.9 | 3.6 ± 1.6 | 2.6 ± 1.2 |
|  | **Session** | 6.8 ± 4.2 | 3.5 ± 1.7 | 3.5 ± 1.5 | 2.5 ± 0.9 |
| **Distance > 90%**  **MSS (m)** | **Set 1** | 78 ± 52 | 63 ± 18 | 33 ± 34 | 17 ± 15 |
|  | **Set 2** | 52 ± 57 | 42 ± 36 | 33 ± 33 | 19 ± 13 |
|  | **Session** | 130 ± 105 | 105 ± 53 | 67 ± 64 | 36 ± 27 |
| **Acceleration load**  **(au)** | **Set 1** | 4.72 ± 3.09 | 2.53 ± 1.37 | 6.25 ± 2.64 | 3.36 ± 1.67 |
|  | **Set 2** | 4.91 ± 2.66 | 2.21 ± 1.20 | 6.86 ± 3.61 | 3.46 ± 1.44 |
|  | **Session** | 9.62 ± 5.63 | 4.75 ± 2.53 | 13.11 ± 6.10 | 6.82 ± 2.87 |
| **RPE-L (au)** | **Set 1** | 47 ± 26 | 42 ± 21 | 34 ± 20 | 19 ± 12 |
|  | **Set 2** | 52 ± 24 | 45 ± 21 | 41 ± 21 | 24 ± 17 |
|  | **Session** | 49 ± 25 | 43 ± 21 | 38 ± 20 | 22 ± 14 |
| **RPE-B (au)** | **Set 1** | 63 ± 22 | 48 ± 20 | 34 ± 16 | 27 ± 14 |
|  | **Set 2** | 73 ± 22 | 58 ± 18 | 41 ± 22 | 28 ± 14 |
|  | **Session** | 68 ± 22 | 53 ± 19 | 37 ± 19 | 28 ± 14 |
| **sRPE-TL (au)** | **Session** | 109 ± 35 | 46 ± 18 | 60 ± 26 | 24 ± 12 |
| Abbreviations: S_best_ = best sprint time; S_avg_ = average sprint time; S_dec_ = percentage sprint decrement; MSS = maximal sprinting speed; avg = average; RPE-L = rating of perceived exertion for the leg muscles; RPE-B = rating of perceived exertion for breathlessness; sRPE-TL = session rating of perceived exertion-training load. | | | | | |

**Supplementary Digital Content 3.** Between protocol comparisons for physiological measures.

| **Outcome** | **Comparison** | **Standardised difference ±90% CL** | ***p_MET_*** |
| --- | --- | --- | --- |
| HR_avg_ | 10×40 vs 5×40 | 0.58; -0.07 to 1.23 | 0.165 |
|  | 10×40 vs 10×20 | 0.69; 0.05 to 1.34 | 0.103 |
|  | 10×40 vs 5×20 | 1.38; 0.74 to 2.03 | 0.002 |
|  | 5×40 vs 10×20 | 0.11; -0.52 to 0.75 | 0.589 |
|  | 5×40 vs 5×20 | 0.80; 0.17 to 1.44 | 0.059 |
|  | 10×20 vs 5×20 | 0.69; 0.05 to 1.33 | 0.101 |
| HR_peak_ | 10×40 vs 5×40 | 0.52; -0.17 to 1.52 | 0.206 |
|  | 10×40 vs 10×20 | 0.72; 0.09 to 1.78 | 0.093 |
|  | 10×40 vs 5×20 | 1.10; 0.60 to 2.26 | 0.011 |
|  | 5×40 vs 10×20 | 0.20; -0.57 to 1.09 | 0.502 |
|  | 5×40 vs 5×20 | 0.58; -0.06 to 1.22 | 0.160 |
|  | 10×20 vs 5×20 | 0.38; -0.26 to 1.02 | 0.316 |
| VO_2avg_ | 10×40 vs 5×40 | 0.18; -0.46 to 0.81 | 0.524 |
|  | 10×40 vs 10×20 | 0.36; -0.27 to 1.00 | 0.333 |
|  | 10×40 vs 5×20 | 0.38; -0.26 to 1.01 | 0.321 |
|  | 5×40 vs 10×20 | 0.19; -0.45 to 0.82 | 0.514 |
|  | 5×40 vs 5×20 | 0.20; -0.43 to 0.83 | 0.501 |
|  | 10×20 vs 5×20 | 0.01; -0.61 to 0.64 | 0.691 |
| T > 90% VO_2max_ | 10×40 vs 5×40 | 1.29; 0.67 to 1.91 | 0.002 |
|  | 10×40 vs 10×20 | 0.88; -0.36 to 2.12 | 0.181 |
|  | 10×40 vs 5×20 | 1.47; 0.84 to 2.11 | <0.001 |
|  | 5×40 vs 10×20 | -0.41; -1.04 to 0.22 | 0.291 |
|  | 5×40 vs 5×20 | 0.18; -0.45 to 0.82 | 0.516 |
|  | 10×20 vs 5×20 | 0.59; -0.04 to 1.23 | 0.151 |
| Abbreviations: HR_avg_ = average heart rate; HR_peak_ = peak heart rate; VO_2avg_ = average oxygen consumption; T > 90% VO_2max_ = time above 90% of VO_2max_; CL = confidence limit | | | |

**Supplementary Digital Content 4.** Between protocol comparisons for perceptual measures.

| **Outcome** | **Comparison** | **Standardised difference ±90% CL** | ***p_MET_*** |
| --- | --- | --- | --- |
| RPE-L | 10×40 vs 5×40 | 0.31; -0.32 to 0.94 | 0.384 |
|  | 10×40 vs 10×20 | 0.59; -0.04 to 1.22 | 0.153 |
|  | 10×40 vs 5×20 | 1.37; 0.74 to 2.00 | 0.001 |
|  | 5×40 vs 10×20 | 0.28; -0.35 to 0.91 | 0.420 |
|  | 5×40 vs 5×20 | 1.06; 0.43 to 1.69 | 0.013 |
|  | 10×20 vs 5×20 | 0.78; 0.15 to 1.41 | 0.063 |
| RPE-B | 10×40 vs 5×40 | 0.79; 0.15 to 1.42 | 0.064 |
|  | 10×40 vs 10×20 | 1.64; 1.01 to 2.28 | <0.001 |
|  | 10×40 vs 5×20 | 2.19; 1.56 to 2.83 | <0.001 |
|  | 5×40 vs 10×20 | 0.85; 0.22 to 1.49 | 0.046 |
|  | 5×40 vs 5×20 | 1.41; 0.77 to 2.04 | 0.001 |
|  | 10×20 vs 5×20 | 0.55; -0.09 to 1.19 | 0.181 |
| sRPE-TL | 10×40 vs 5×40 | 2.59; 1.96 to 3.23 | <0.001 |
|  | 10×40 vs 10×20 | 2.00; 1.37 to 2.63 | <0.001 |
|  | 10×40 vs 5×20 | 3.47; 2.84 to 4.11 | <0.001 |
|  | 5×40 vs 10×20 | -0.59; -1.23 to 0.04 | 0.152 |
|  | 5×40 vs 5×20 | 0.88; 0.25 to 1.51 | 0.039 |
|  | 10×20 vs 5×20 | 1.47; 0.84 to 2.11 | <0.001 |
| Abbreviations: RPE-L = differential rating of perceived exertion for the leg muscles; RPE-B = differential rating of perceived exertion for breathlessness; sRPE-TL = session rating of perceived exertion-training load; CI = confidence limit | | | |

**Supplementary Digital Content 5.** Between protocol comparisons for performance measures.

| **Outcome** | **Comparison** | **Standardised difference ±90% CL** | ***p_MET_*** |
| --- | --- | --- | --- |
| S_dec_ | 10×40 vs 5×40 | 1.37; 0.74 to 2.01 | 0.002 |
|  | 10×40 vs 10×20 | 1.39; 0.75 to 2.03 | 0.001 |
|  | 10×40 vs 5×20 | 1.79; 1.16 to 2.43 | <0.001 |
|  | 5×40 vs 10×20 | 0.02; -0.62 to 0.66 | 0.682 |
|  | 5×40 vs 5×20 | 0.42; -0.22 to 1.06 | 0.282 |
|  | 10×20 vs 5×20 | 0.40; -0.23 to 1.04 | 0.297 |
| Distance > 90% MSS | 10×40 vs 5×40 | 0.37; -0.26 to 1.01 | 0.323 |
|  | 10×40 vs 10×20 | 0.94; 0.30 to 1.57 | 0.029 |
|  | 10×40 vs 5×20 | 1.38; 0.75 to 2.02 | 0.001 |
|  | 5×40 vs 10×20 | 0.56; -0.07 to 1.20 | 0.172 |
|  | 5×40 vs 5×20 | 1.01; 0.38 to 1.64 | 0.018 |
|  | 10×20 vs 5×20 | 0.45; -0.19 to 1.08 | 0.258 |
| Acceleration load | 10×40 vs 5×40 | 1.07; 0.43 to 1.70 | 0.013 |
|  | 10×40 vs 10×20 | -0.76; -1.40 to -0.13 | 0.071 |
|  | 10×40 vs 5×20 | 0.61; -0.02 to 1.25 | 0.140 |
|  | 5×40 vs 10×20 | -1.83; -2.46 to -1.20 | <0.001 |
|  | 5×40 vs 5×20 | -0.45; -1.09 to 0.18 | 0.252 |
|  | 10×20 vs 5×20 | 1.38; 0.74 to 2.01 | 0.002 |
| Abbreviations: S_dec_ = percentage sprint decrement; MSS = maximal sprint speed; CL = confidence limit | | | |

**Supplementary Digital Content 6.** The time course of recovery of neuromuscular performance within each repeated-sprint training protocol.

|  | **Pre**  **(mean ±SD)** | **Pre-post** | | |  | **Pre-24 hr** | | |  | **Pre-48 hr** | | |
| --- | --- | --- | --- | --- | --- | --- | --- | --- | --- | --- | --- | --- |
|  |  | **Change**  **±90% CI** | **Standardised**  **difference**  **±90 CI** | ***p_MET_*** |  | **Change**  **±90% CI** | **Standardised**  **difference**  **±90 CI** | ***p_MET_*** |  | **Change**  **±90% CI** | **Standardised**  **difference**  **±90 CI** | ***p_MET_*** |
| **Hamstring PF90° (N)** | | | | | | | | | | | | |
| 10×40 | 235 ±54 | -10 ±34 | -0.18 ±0.63 | 0.52 |  | -8 ±34 | -0.16 ±0.63 | 0.55 |  | -4 ± 34 | -0.08 ±0.63 | 0.62 |
| 5×40 | 227 ±58 | -8 ±32 | -0.16 ±0.63 | 0.54 |  | -2 ±32 | -0.04 ±0.63 | 0.66 |  | -5 ±32 | -0.11 ±0.63 | 0.60 |
| 10×20 | 226 ±48 | -2 ±32 | -0.03 ±0.64 | 0.67 |  | 8 ±32 | 0.16 ±0.63 | 0.54 |  | 11 ±32 | 0.22 ±0.65 | 0.48 |
| 5×20 | 224 ±50 | -2 ±32 | -0.04 ±0.53 | 0.70 |  | -6 ±33 | -0.11 ±0.63 | 0.59 |  | 5 ±32 | -0.03 ±0.64 | 0.61 |
|  |  |  |  |  |  |  |  |  |  |  |  |  |
| **Hamstring PF30° (N)** | | | | | | | | | | | | |
| 10×40 | 227 ±61 | -4 ±36 | -0.07 ±0.66 | 0.63 |  | -4 ±36 | -0.07 ±0.66 | 0.63 |  | -3 ±36 | -0.05 ±0.66 | 0.64 |
| 5×40 | 220 ±54 | -8 ±31 | -0.15 ±0.62 | 0.56 |  | -1 ±31 | -0.02 ±0.61 | 0.69 |  | -2 ±31 | -0.03 ±0.62 | 0.67 |
| 10×20 | 223 ±50 | 4 ±30 | 0.08 ±0.60 | 0.63 |  | 5 ±30 | 0.09 ±0.60 | 0.62 |  | 0 ±67 | 0.00 ± 1.32 | 0.60 |
| 5×20 | 221 ±50 | 0 ±32 | 0.00 ±0.63 | 0.70 |  | 2 ±34 | 0.03 ±0.67 | 0.66 |  | 6 ±33 | 0.11 ±0.64 | 0.59 |
|  |  |  |  |  |  |  |  |  |  |  |  |  |
| **CMJ height (cm)** | | | | | | | | | | | | |
| 10×40 | 35.9 ±7.1 | -1.1 ±4.5 | -0.15 ±0.63 | 0.55 |  | -1.4 ±4.5 | -0.20 ±0.63 | 0.50 |  | 0.0 ±4.4 | -0.01 ±0.61 | 0.70 |
| 5×40 | 35.7 ±7.7 | 0.4 ±4.8 | 0.05 ±0.63 | 0.65 |  | -0.3 ±4.8 | -0.04 ±0.64 | 0.66 |  | 0.0 ±4.7 | 0.00 ±0.62 | 0.70 |
| 10×20 | 35.9 ±7.4 | -0.2 ±4.7 | -0.03 ±0.64 | 0.67 |  | -0.7 ±4.7 | -0.09 ±0.64 | 0.61 |  | 0.4 ±4.8 | 0.05 ±0.65 | 0.65 |
| 5×20 | 36.1 ±7.7 | -0.6 ±4.9 | -0.08 ±0.63 | 0.62 |  | -1.3 ±5.0 | -0.17 ±0.64 | 0.53 |  | 0.3 ±4.9 | 0.04 ±0.63 | 0.66 |
|  |  |  |  |  |  |  |  |  |  |  |  |  |
| **CMJ mean power (W.kg)** | | | | | | | | | | | | |
| 10×40 | 25.8 ±4.3 | -0.6 ±2.8 | -0.13 ±0.63 | 0.57 |  | -0.9 ±2.8 | -0.20 ±0.63 | 0.50 |  | 0.4 ±2.8 | 0.08 ±0.63 | 0.62 |
| 5×40 | 26.0 ±4.2 | 0.5 ±2.7 | 0.12 ±0.63 | 0.58 |  | -0.4 ±2.7 | -0.10 ±0.63 | 0.61 |  | -0.3 ±2.7 | -0.06 ±0.63 | 0.64 |
| 10×20 | 25.8 ±4.6 | 0.6 ±2.9 | 0.12 ±0.63 | 0.58 |  | -0.1 ±2.9 | -0.03 ±0.64 | 0.67 |  | 0.2 ±3.0 | 0.05 ±0.65 | 0.65 |
| 5×20 | 25.6 ±4.6 | 0.4 ±3.0 | 0.09 ±0.63 | 0.61 |  | -0.6 ±2.9 | -0.12 ±0.88 | 0.59 |  | 0.6 ±4.2 | 0.12 ±0.88 | 0.56 |
| Continued next page |  |  |  |  |  |  |  |  |  |  |  |  |
| **CMJ FT:CT** | | | | | | | | | | | | |
| 10×40 | 0.58 ±0.13 |  |  |  |  |  |  |  |  |  |  |  |
| 5×40 | 0.59 ±0.10 |  |  |  |  |  |  |  |  |  |  |  |
| 10×20 | 0.58 ±0.14 |  |  |  |  |  |  |  |  |  |  |  |
| **CMJ FT:CT** |  |  |  |  |  |  |  |  |  |  |  |  |
| 10×40 | 0.58 ±0.13 | 0.00 ±0.09 | -0.02 ±0.66 | 0.67 |  | -0.01 ±0.08 | -0.08 ±0.63 | 0.63 |  | 0.03 ±0.08 | 0.19 ±0.63 | 0.51 |
| 5×40 | 0.59 ±0.10 | 0.03 ±0.07 | 0.29 ±0.64 | 0.40 |  | -0.01 ±0.08 | -0.09 ±0.63 | 0.62 |  | 0.01 ±0.07 | 0.11 ±0.62 | 0.60 |
| 10×20 | 0.58 ±0.14 | 0.04 ±0.09 | 0.27 ±0.64 | 0.43 |  | 0.00 ±0.08 | 0.03 ±0.60 | 0.68 |  | 0.01 ±0.09 | 0.06 ±0.62 | 0.64 |
| 5×20 | 0.57 ±0.15 | 0.03 ±0.08 | 0.22 ±0.63 | 0.48 |  | 0.00 ±0.08 | -0.03 ±0.63 | 0.67 |  | 0.01 ±0.09 | 0.10 ±0.64 | 0.61 |
|  | | | | | | | | | | | | |
| **CMJ EccDur** | | | | | | | | | | | | |
| 10×40 | 667 ±199 | -15 ±99 | -0.10 ±0.63 | 0.61 |  | -11 ±100 | -0.07 ±0.64 | 0.63 |  | 55 ±99 | -0.35 ±0.63 | 0.35 |
| 5×40 | 642 ±120 | -36 ±111 | -0.20 ±0.63 | 0.44 |  | 7 ±112 | 0.04 ±0.63 | 0.61 |  | -8 ±113 | -0.04 ±0.64 | 0.61 |
| 10×20 | 660 ±164 | -70 ±102 | -0.53 ±0.78 | 0.25 |  | -24 ±102 | -0.18 ±0.78 | 0.53 |  | -20 ±104 | -0.15 ±0.79 | 0.55 |
| 5×20 | 672 ±196 | -47 ±90 | -0.34 ±0.64 | 0.20 |  | -32 ±92 | -0.23 ±0.65 | 0.29 |  | 29 ±90 | -0.20 ±0.64 | 0.31 |
|  |  |  |  |  |  |  |  |  |  |  |  |  |
| **Leg stiffness** | | | | | | | | | | | | |
| 10×40 | 43.9 ±7.4 | -2.0 ±5.1 | -0.25 ±0.63 | 0.45 |  | -2.0 ±5.1 | -0.25 ±0.63 | 0.45 |  | -0.3 ±5.1 | -0.04 ±0.63 | 0.67 |
| 5×40 | 43.0 ±8.2 | -1.2 ±5.1 | -0.15 ±0.63 | 0.55 |  | -0.8 ±5.1 | -0.09 ±0.63 | 0.61 |  | 0.2 ±5.1 | 0.02 ±0.63 | 0.68 |
| 10×20 | 42.9 ±8.8 | -0.3 ±5.3 | -0.04 ±0.63 | 0.66 |  | 1.6 ±5.3 | 0.20 ±0.63 | 0.50 |  | 0.7 ±5.4 | 0.09 ±0.64 | 0.62 |
| 5×20 | 42.6 ±8.5 | 0.1 ±5.6 | 0.01 ±0.62 | 0.69 |  | 0.5 ±5.8 | 0.05 ±0.64 | 0.65 |  | 1.5 ±5.7 | 0.16 ±0.63 | 0.54 |
|  |  |  |  |  |  |  |  |  |  |  |  |  |
| Abbreviations: PF90° = peak force at 90° of knee flexion; PF30° = peak force at 30° of knee flexion; CMJ = countermovement jump; FT:CT = flight-time to contraction-time ratio; EccDur = eccentric duration; SD = standard deviation; CI = confidence interval; hr = hour. | | | | | | | | | | | | |
